# Core bacterial community composition of a cryptoendolithic ecosystem in the Grand Staircase‐Escalante National Monument, Utah

**DOI:** 10.1002/mbo3.707

**Published:** 2018-08-05

**Authors:** Sukhpreet Kaur, HD Kurtz

**Affiliations:** ^1^ Department of Biological Sciences Clemson University Clemson South Carolina

**Keywords:** *Acidiphilium* spp, amplicon sequencing, cryptoendolithic bacterial communities, *Cyanobacteria*, GSENM

## Abstract

Cryptoendolithic bacterial communities in the Jurassic Navajo Sandstones play an important ecological role in this ecosystem. Developing a better understanding of the role of these cryptoendolithic communities required a deeper knowledge of the microbial diversity present. We analyzed the bacterial diversity in eight sandstones samples from several microgeological features associated with a large sandstone dome. Cryptoendolithic bacterial diversity is clustered into three distinct groups which correlated with topography, suggesting the duration of water retention might be a factor. Comparisons of diversity between each cluster showed that a core bacterial community exists in this habitat. The overall bacterial community structure was dominated by *Cyanobacteria*,* Proteobacteria*,* Bacteroidetes,* and *Actinobacteria*. The most prevalent genera in cyanobacteria were *Leptolyngbya*,* Chroococcidiopsis,* and unclassified cyanobacteria accounting for the bulk of cyanobacterial sequences. Within the *Proteobacteria*, Alphaproteobacteria were the largest class detected, with members of the Acetobacteraceae, particularly the genus *Acidiphilium*, being the most abundant. *Acidiphilium* spp. are capable of aerobic ferric iron reduction under moderately acidic conditions, explaining the high levels of iron (II) in this system. This study highlights the extent of unexplored bacterial diversity in this habitat system and sets the premise for elaborating on the ecological function of cryptoendolithic communities.

## 1 INTRODUCTION

Deserts constitute the most extensive terrestrial biome and cover about 30% of the United States land area (Housman, Powers, Collins, & Belnap, 2006; Pointing & Belnap, 2012). The Grand Staircase‐Escalante National Monument (GSENM) located in southern Utah, encompasses a large area of semiarid and arid regions with diverse geological features (Doelling, Blackett, Hamblin, Powell, & Pollock, 2000). Throughout the GSENM, large sandstone domes and other barren rock outcrops dominate the landscape with expanses of rocky flats interspersed between elevated features. This is especially prevalent in the arid Escalante Canyons region of the monument. Amongst the different rock formations in GSENM, the eolian Jurassic Navajo sandstone unit is known for patterns of pink to red coloration resulting from iron removal throughout its burial history (Beitler, Parry, & Chan, 2005; Potter‐McIntyre et al., 2013). Some areas of the Jurassic Navajo sandstone have been bleached of its color, which has been mainly attributed to paleogeochemical processes (Beitler, Chan, & Parry, 2003; Chan, Parry, & Bowman, 2000; Potter & Chan, 2011). Previous studies suggest that microorganisms are at least partially involved in this ongoing process of iron bleaching (Hammes, Floyd, & Kurtz, 2013; Loope, Kettler, & Weber, 2010).

The Jurassic Navajo Sandstone unit is a friable, poorly cemented sandstone that is highly porous (Kurtz & Netoff, 2001). Moisture availability is a major constraint affecting microbial diversity and activity in arid environments (Bhatnagar & Bhatnagar, 2005; Laity, 2009; Potts & Friedmann, 1981). The irregular system of pores in sandstones provides a protective network for microorganisms, creating a place for condensation and retention of water, while allowing light to penetrate the upper sandstone surface (Bell, 1993; Friedmann, 1980; Walker & Pace, 2007). In hot deserts, the combined effects of temperature and aridity along with lack of nutrients in sandstones leads to unique adaptations in desert microbiota (Gorbushina, 2007; Makhalanyane et al., 2015).

One survival adaptation of bacterial communities is to reside within the pore spaces of desert sandstones as cryptoendoliths. This ecological niche provides microorganisms physical stability and protection from extreme environmental conditions in hot deserts (Archer et al., 2017; Friedmann & Ocampo, 1976; Wierzchos, de los Ríos, & Ascaso, 2012; Yung et al., 2014). Cryptoendolithic communities produce extracellular polymeric substances (EPS) under moist conditions as another survival adaptation to retain water, entrap nutrients, and reduce temperature fluctuations (Antony, Cockell, & Shouche, 2012; Büdel et al., 2004; Gorbushina & Broughton, 2009; Kurtz, 2002; Lacap‐Bugler et al., 2017; Omelon, Pollard, & Ferris, 2006). It has also been observed that cryptoendolithic biomass acts to stabilize the friable sandstone surface through the production of EPS and in some cases by filamentous cell growth (Kurtz & Cox, 2010), protecting the sandstone surfaces from erosional processes, resulting in diverse microscale features, such as rock visors and undercut ripples in the sandstones (Kurtz & Netoff, 2001). Such microgeomorphological features have a direct effect on water and nutrient availability based on infiltration, runoff, erosion, and water accumulation (Li, He, Zerbe, Li, & Liu, 2010). This geologic heterogeneity increases the potential for heterogeneous communities being assembled in the cryptoendolithic habitat.

It is well known that cyanobacteria are the primary producers within the cryptoendolithic communities, providing energy in the form of fixed carbon by photosynthesis and supporting the growth of heterotrophic microorganisms (Archer et al., 2017; Casamatta, Verb, Beaver, & Vis, 2002; de la Torre, Goebel, Friedmann, & Pace, 2003). Cryptoendolithic bacterial communities are involved in nutrient cycling within the oligotrophic rock substratum (Hammes et al., 2013; Kurtz, Cox, & Reisch, 2005). Microbial diversity within these sandstones has previously been studied using microscopic and molecular techniques (Bell, 1993; Hammes et al., 2013; Kurtz et al., 2005). While these analyses have provided some data on the structure of these cryptoendolithic communities, the data are insufficient to accurately describe these communities. To address the lack of depth with respect to cryptoendolithic community structure in the Jurassic Navajo sandstones, we used Illumina MiSeq sequencing technology to acquire the requisite data.

In this study, we examine the bacterial diversity of cryptoendolithic communities associated with the Jurassic Navajo sandstone of the GSENM. Considering the heterogeneity of geological features around this landform, we expect that the subcommunities would differ from each other and that, from these data, a core bacterial community could be derived. Additionally, we expect to find taxa having members known to participate in nutrient cycling. Below, we provide data to support these hypotheses.

## 2 MATERIALS AND METHODS

### 2.1 Site characterization and sampling procedure

**Figure 1:**
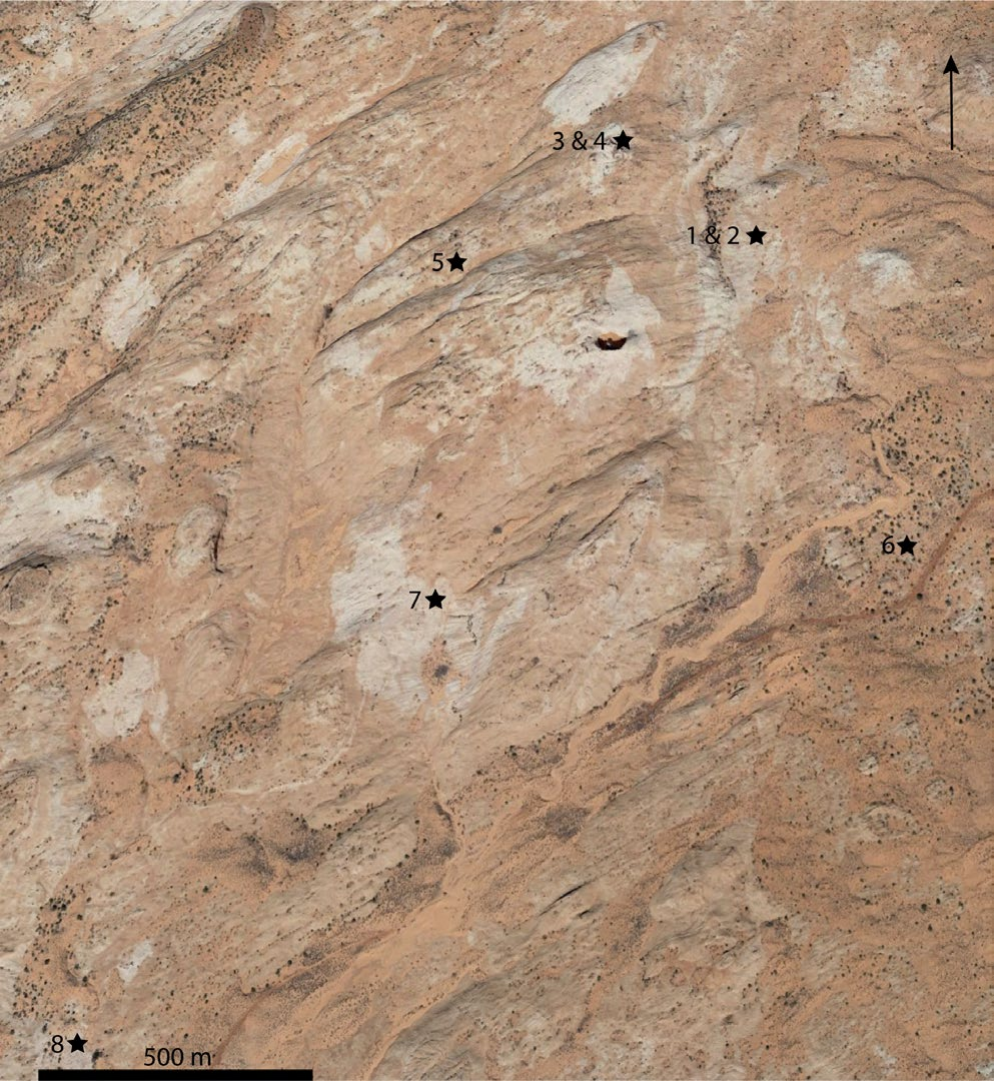
Map of sampling sites in the Harris Wash area of the GSENM obtained through Google Earth. Sandstones corresponding to sampling sites and the GPS coordinates are shown in Table 1. Scale bar in the lower right corner equals 500 m. Arrows indicates north

**Figure 2:**
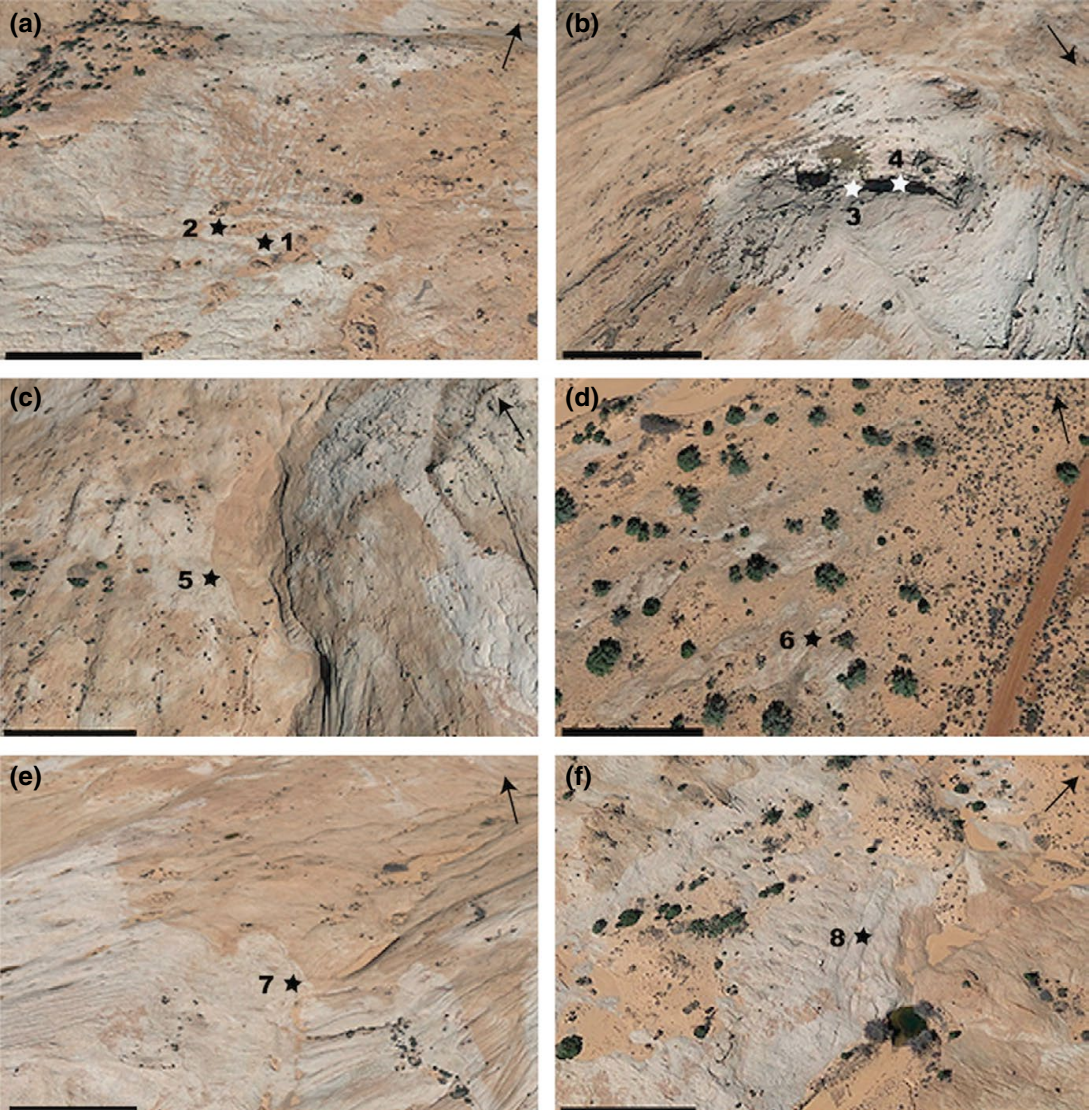
Close‐up view of the sampling sites obtained through Google Earth, representing (a) slickrock HW01_04 and slickrock HW03_04 associated with sediments; (b) Samples HW04_04 and HW06_04 from an alcove area represented by the darker colored region; (c) slickrock HW07_04 obtained from a slope; (d, e) slickrock HW01_05 and slickrock HW04_05 associated with sediments; and (f) slickrock HW07_05. Scale bars in the bottom left corner represent 35 m. The arrows indicate north direction

All samples were obtained from the Jurassic Navajo Sandstone, an eolian sandstone unit located in the Harris Wash area of the GSENM in southern Utah (Hammes et al., 2013). Eight sandstone samples were collected from sites around a sandstone dome having different topographic features (Table 1, Figure 1). The HW in the samples denotes the Harris Wash area followed by the site number and the year of sampling. Three samples, HW01_04, HW01_05, and HW07_05 were from rock surfaces that were slightly sloped and without eolian sediments. HW07_04 was obtained from a rock slope (Figure 2). HW03_04 and HW04_05 were from rock surfaces near sediment deposits near the base of a sandstone dome. Two samples, HW06_04 and HW04_04, were obtained from an alcove that is a wind‐eroded depression in a small cliff (Figure 2). Samples were obtained using a chisel to remove the upper 5–10 mm of sandstone surface from an area of approximately 50 cm^2^ and placed into sterile sample bags. All the sandstone samples were stored in the dark at room temperature as dry samples until further processing.

**Table 1:** Sampling sites of the eight Jurassic Navajo sandstones analyzed in this study

Site	Sandstone	Sampling year	Location	Notes
1	HW01_04	2004	37° 41′ 10.02″ N; 111° 18′ 41.70″ W	Slick rock
2	HW03_04	2004	37° 41′ 10.02″ N; 111° 18′ 41.70″ W	Associated with sediments
3	HW04_04	2004	37° 41′ 17.73″ N; 111° 18′ 56.74″ W	Alcove area
4	HW06_04	2004	37° 41′ 17.73″ N; 111° 18′ 56.74″ W	Alcove area
5	HW07_04	2004	37° 41′ 06.69″ N; 111° 19′ 11.64″ W	Slick rock slope
6	HW01_05	2005	37° 40′ 42.36″ N; 111° 18′ 29.28″ W	Slick rock
7	HW04_05	2005	37° 40′ 38.80″ N; 111° 18′ 11.23″ W	Associated with sediments
8	HW07_05	2005	37° 40′ 08.49″ N; 111° 19′ 37.81″ W	Slick rock

### 2.2 Chemical analysis of the sandstones

Sandstone color was analyzed in comparison with the Munsell Soil Color Charts (Munsell Color Company, 1975) assigning each moist sandstone sample to the nearest integer unit of hue, value, and chroma (Escadafal, Girard, & Courault, 1989). The pH of the sandstone samples was measured with an Accumet Research AR 25 dual channel pH/ion meter (Thermo Fisher Scientific Ltd, USA) using the slurry technique, by mixing 1 g of crushed sandstone with 2.5 ml of deionized water and allowing the samples to settle (Lee, Barbier, Bottos, McDonald, & Cary, 2012). Nitrate, ammonium, nitrite, sulfate, and ferrous ion levels were measured using colorimetric assays following methods that have been described earlier (Carter, 1971; Gerhardt, 1994; Kartal et al., 2006; Souza et al., 2012). Phosphate levels were determined using the Malachite Green Phosphate Assay Kit (Cayman Chemical, USA) based on a colorimetric assay (D'Angelo, Crutchfield, & Vandiviere, 2001).

### 2.3 DNA extraction and Illumina 16S rRNA amplicon sequencing

Total genomic DNA was extracted from approximately 500 mg of each sandstone sample using the PowerSoil^®^ DNA Isolation Kit (Mo Bio Laboratories Inc., USA) following the manufacturer's instructions. Ten nanograms of DNA from each sample was used to amplify the V4 region of the 16S rRNA genes following the methods as listed in Schloss MiSeq Wet Lab standard operating procedures (Kozich, Westcott, Baxter, Highlander, & Schloss, 2013). The amplified PCR products were then submitted to the Duke Genome Sequencing and Analysis Core Facility for Illumina MiSeq sequencing.

### 2.4 Bioinformatics analysis

Illumina sequence reads were processed using the Mothur software package, version 1.39.1 (Schloss et al., 2009). Contiguous sequences (contigs) were created by merging the forward and reverse sequences using mothur pipeline. The Ribosomal Database Project (RDP) pipeline was used to trim the ends of the sequences to 255 base pairs so that all the sequences started and ended at the same coordinates (Cole et al., 2009). All further analysis was performed using mothur following the MiSeq standard operating procedures (Kozich et al., 2013). Processed sequences were screened for chimeras using the UCHIME algorithm within mothur (Edgar, Haas, Clemente, Quince, & Knight, 2011). All sequences were classified using the Bayesian classifier against the SILVA database (Pruesse et al., 2007) and clustered into operational taxonomic units (OTUs) using the average neighbor‐joining method at 97% identity followed by taxonomy assignment.

To account for differences in the number of sequences for each sample, the dataset was rarefied by subsampling to the smallest sample dataset with 13041 sequences using mothur (Schloss et al., 2009). Chao1 richness indicators and inverse Simpson diversity indices were used to assess bacterial richness and evenness. Dendrograms were constructed to describe the similarity between the sandstone samples at phyla, hierarchical level, based on thetayc coefficients using mothur pipeline (Kozich et al., 2013). Principal coordinate analysis plots were constructed using an eigenvector‐based approach using thetayc calculator to examine the bacterial community OTU relatedness between the sandstone samples, the more related samples tend to be clustered together. The variability between the clusters was tested using the analysis of molecular variance (AMOVA) statistical method in the mothur pipeline (Schloss et al., 2009). The OTUs responsible for the spatial separation of microbial communities along the two axes of the PCoA plot were measured by the correlation of the relative abundance of each OTU with the two axes using the nonparametric Spearman correlation method. The cumulative diversity within each cluster was compared to obtain a core bacterial community that was shared between the clusters using Venn diagram analysis. To test for any correlation between the relative OTU abundance data for each sandstone sample and physiochemical parameters, we constructed the canonical correspondence analysis plot using the eigenanalysis algorithm in the Paleontological Statistics Software, version 3.19 (Hammer, Harper, & Ryan, 2001; Legendre & Legendre, 2012).

### 2.5 Sequence data availability

Fastq files containing the raw data from this study were submitted to the NCBI sequence read archive (www.ncbi.nlm.nih.gov/sra) and can be accessed by the BioProject number PRJNA292826.

## 3 RESULTS

### 3.1 Chemical analysis of the sandstone samples

The pH of all the sandstone samples was fairly constant ranging between 6.4 and 6.8 (Table 2). Nitrite seemed to be completely absent in two samples, namely HW07_04 and HW04_04, while in the other samples, it ranged between 0.4 and 5.4 nanomoles/g dry weight of sandstone. Nitrate levels ranged between 21 and 660 nanomoles/g dry weight of sandstone, while ammonium levels ranged between 212 and 4000 nanomoles/g dry weight of sandstone. Phosphate and sulfate levels measured were within 112–472 and 0.4–23.9 nanomoles/g dry weight of sandstone, respectively. Ferrous iron concentrations ranged between 98 and 280 nanomoles/g dry weight of sandstone. The sandstone color was in the spectrum of reddish brown‐pink to pale red. The Munsell color notation for moist sandstone samples showed that sandstone color was fairly uniform in all the sandstone samples, the hue was 2.5–5 year, and the value and chroma ranged between 4/8 and 8/4 as shown in Table 2. The canonical correspondence analysis (CCA) plot showed no correlation between the relative OTU abundance and the physiochemical parameters of the sandstone samples (data not shown).

**Table 2:** Physiochemical analysis of the Jurassic Navajo sandstone samples

Sandstones	Nitrate^a^	Nitrite^a^	Ammonium^a^	Sulfate^a^	Phosphate^a^	Ferrous iron^a^	Munsell color (wet)	pH
HW01_04	232.94	2	950.77	0.38	228.70	149.39	2.5 year 8/4	6.36
HW03_04	291.76	0.4	966.15	6.86	196.73	170.26	2.5 year 5/4	6.57
HW04_04	366.27	0	4007.18	9.56	472.14	165.04	2.5 year 5/6	6.36
HW06_04	21.18	2.6	381.54	23.88	456.06	143.59	2.5 year 4/8	6.52
HW07_04	134.90	2	1627.69	6.59	317.27	279.54	5 year 5/3	6.49
HW01_05	397.65	5.4	212.31	21.44	332.51	122.14	2.5 year 7/4	6.80
HW04_05	350.59	2.2	458.46	1.73	160.43	98.38	2.5 year 8/3	6.51
HW07_05	660.39	0	268.72	6.32	126.96	153.74	2.5 year 7/2	6.69

a The units are nanomoles per gram of dry weight of crushed sandstone.

### 3.2 Overview of the total bacterial diversity in cryptoendolithic communities

**Figure 3:**
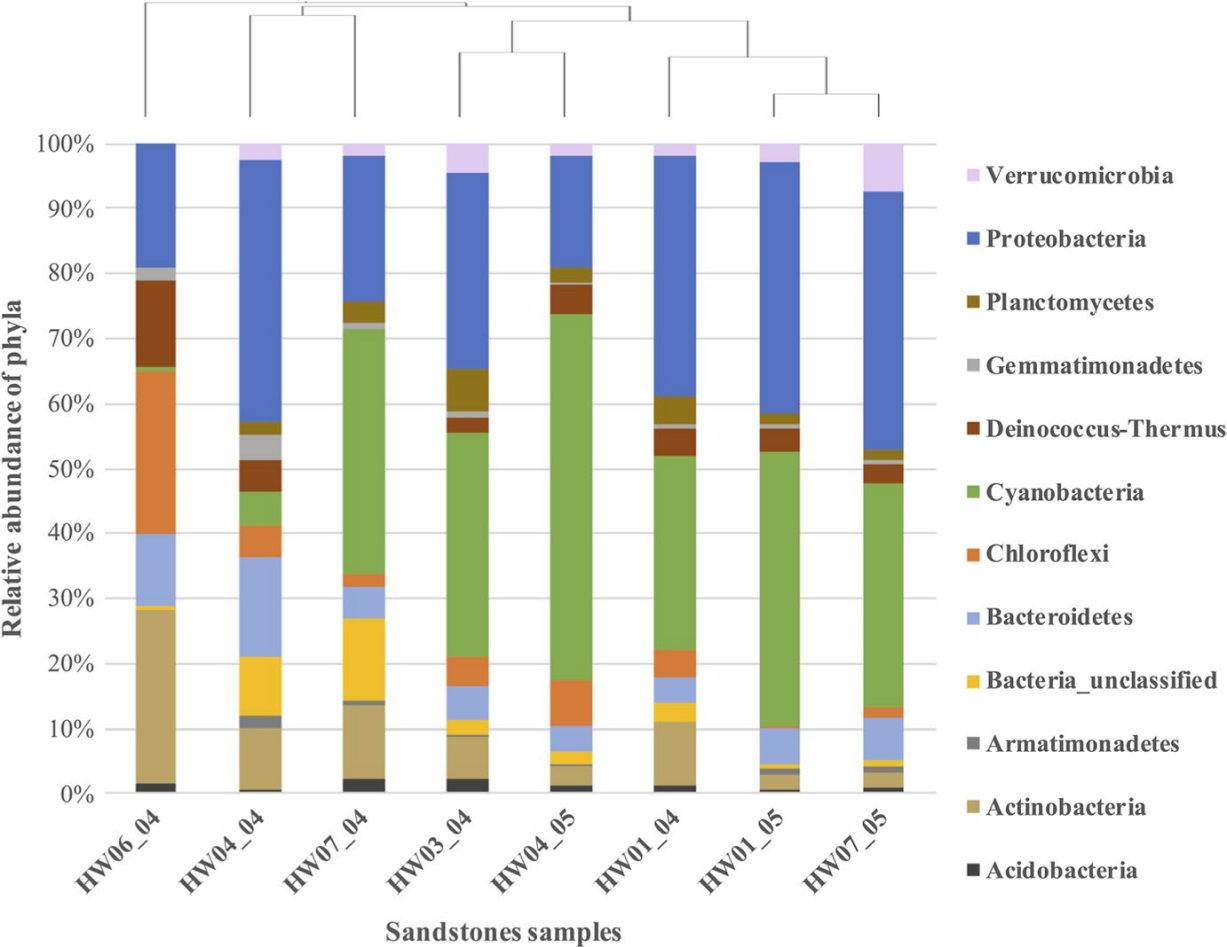
Relative abundances of the major phyla identified with >0.1% sequence abundance in cryptoendolithic bacterial communities. Taxa are arranged in order as they appear on the stacked bar graph with each rectangle representing the relative percentage abundance of a phylum in a particular sandstone sample

Sequences from the eight sandstone samples were pooled and processed together, resulting in a total of 152,451 high‐quality sequences with an average length of 253 bases. A total of 2,487 OTUs were generated after clustering at a 97% similarity index. Relative percentage abundances of the taxa observed in each sandstone sample were calculated using the number of sequences obtained for each taxon against the total number of sequences obtained for that particular sandstone sample. Phyla with greater than 0.1% sequence abundance were analyzed, resulting in 12 distinct phyla observed amongst all sandstone samples (Figure 3).

Based on the relative abundance of phyla in all sandstone samples, 35% of the total sequences were assigned to *Cyanobacteria* making it the most abundant phylum (Supporting Information Table S1). *Cyanobacteria* were prominent in all samples except the sandstone samples collected from alcove region, HW04_04 and HW06_04 (Figure 3). The most abundant OTUs belonged to the genus *Leptolyngbya* followed by unclassified cyanobacteria and *Mastigocladopsis* (Supporting Information Table S2).

The second most abundant phylum was *Proteobacteria* represented by 28.5% of the total bacterial sequences obtained from all sandstone samples (Supporting Information Table S1). Within the phylum *Proteobacteria*, 95% sequences belonged to the class Alphaproteobacteria. The genus *Acidiphilium,* a member of the family Acetobacteraceae, was the most abundant OTU in this class (Supporting Information Table S2).

*Actinobacteria* was the third most abundant phylum with 7.8% of the total sequences followed by *Bacteroidetes* with 7.4% of the total sequences. When considering the sequences with greater than 0.1% relative abundance, all of the sequences assigned to *Actinobacteria* were binned to unclassified families and genera. *Bacteroidetes* had the highest number of bacteria in class Sphingobacteria, family Chitinophagaceae, and genus *Segetibacter*. About 1.5%–5.5% of the total sequences were assigned to other phyla that included *Chloroflexi, Deinococcus–Thermus, Verrucomicrobia, Planctomycetes, and Gemmatimonadetes* (Figure 3). For the all sandstone samples analyzed, approximately 4.1% of the total sequences were binned to unclassified phyla.

Comparing the bacterial diversity between sandstone samples at phyla level based on the dendrogram suggests that slick rocks HW01_04, HW01_05, and HW07_05 are more related to each other, while HW03_04 and HW04_05 (slick rock associated with sediments) formed another clade (Figure 3). HW04_04 and HW07_04 (alcove and slick rock slope sample) are more related, while HW06_04 seems to differ from the rest of the sandstone samples (Figure 3). *Proteobacteria* were the most abundant in HW04_04, while *Actinobacteria* were the most abundant in HW06_04 and HW7_04 samples (Supporting Information Table S1). *Bacteroidetes* were present in greater numbers in the alcove samples (HW04_04 and HW06_04) compared to the other sandstone samples. *Deinococcus‐Thermus* and *Chloroflexi* were notably present in higher numbers in HW06_04, while *Verrucomicrobia* numbers declined to less than 0.1% (Supporting Information Table S1). *Planctomycetes* and *Verrucomicrobia* were present in greater numbers in the slick rock samples compared to the alcove samples. Unclassified phyla accounted for 12.5% of the sequences obtained from HW07_04, the sandstone sample collected from a slope (Supporting Information Table S1).

### 3.3 Analysis of cyanobacterial community structure

Based on the relative abundance of sequences, at the order of hierarchy level, Cyanobacteria_Subsection III was the most dominant comprising 13% sequences, with unclassified Cyanobacteria accounting for 8.6% of total sequences. Cyanobacteria_Subsection II was the third most abundant order followed by Cyanobacteria_Subsection I, each spanning 6.64% and 5.29% of the total sequences, respectively. At the family level, 34% of the total sequences were assigned to Cyanobacteria_Subsection III_FamilyI, unclassified Cyanobacteria, Cyanobacteria_Subsection II _FamilyII, and Cyanobacteria_SubsectionI_FamilyI. Cumulatively, at the genus level, *Leptolyngbya* was the most abundant genus occupying 9.4% sequences followed by unclassified cyanobacteria, *Chroococcidiopsis, Mastigocladopsis,* and Cyanobacteria_SubsectionIII_Family_unclassified occupying 8.7%, 6.6%, 5.3%, and 3.7% of the total sequences, respectively. Other genera included *Nostoc*,* Synechococcus,* and *Microcoleus* each representing less than 1% of the overall cyanobacterial diversity detected in all sandstone samples analyzed.

**Figure 4:**
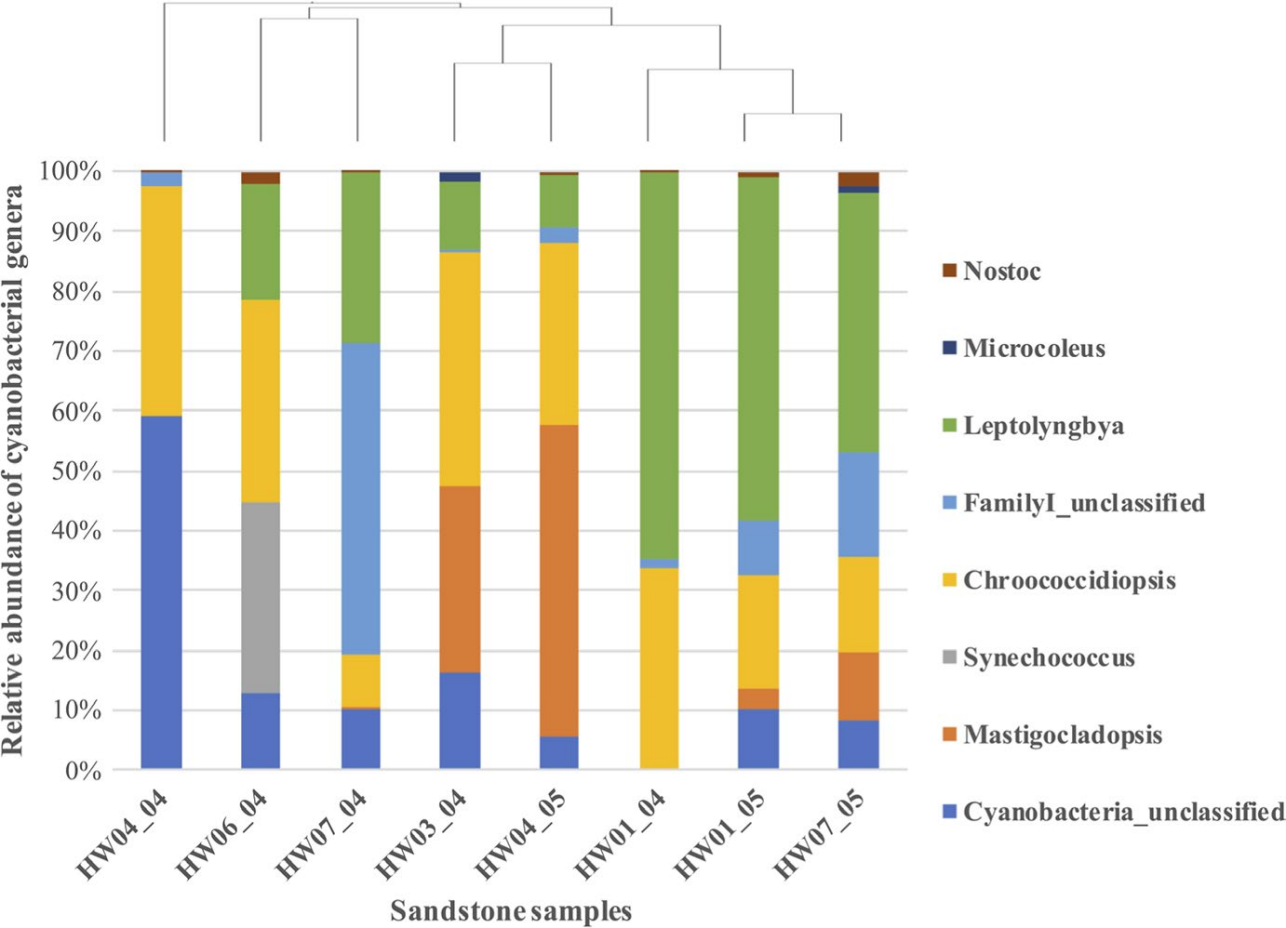
Comparison of the relative abundance of cryptoendolithic cyanobacterial communities in different sandstone samples. Taxa are arranged in order as they appear on the stacked bar graph with each rectangle representing the relative percentage of cyanobacterial genera in a particular sandstone sample

Broadly, *Leptolyngbya* and *Chroococcidiopsis* were the most prevalent genera in all sandstone samples tested except HW04_04 in which the former was completely absent (Figure 4). *Synechococcus* had less than 0.1% relative abundance in all samples except one of the alcove samples, HW06_04, where it occupied 32% of the total cyanobacterial sequences at genera level. Cyanobacteria_SubsectionIII_Family_unclassified occupied 52% of the total cyanobacterial sequences in the slick rock slope sample, HW07_04, while unclassified cyanobacteria spanned 59.2% of the cyanobacterial sequences in one alcove sample, HW04_04. *Mastigocladopsis* was present in higher numbers in HW03_04 and HW04_05, which were stone samples taken near eolian sediment deposits, while this genus was completely absent in one of the alcove samples, HW06_04 (Figure 4).

### 3.4 Bacterial species richness and diversity

**Figure 5:**
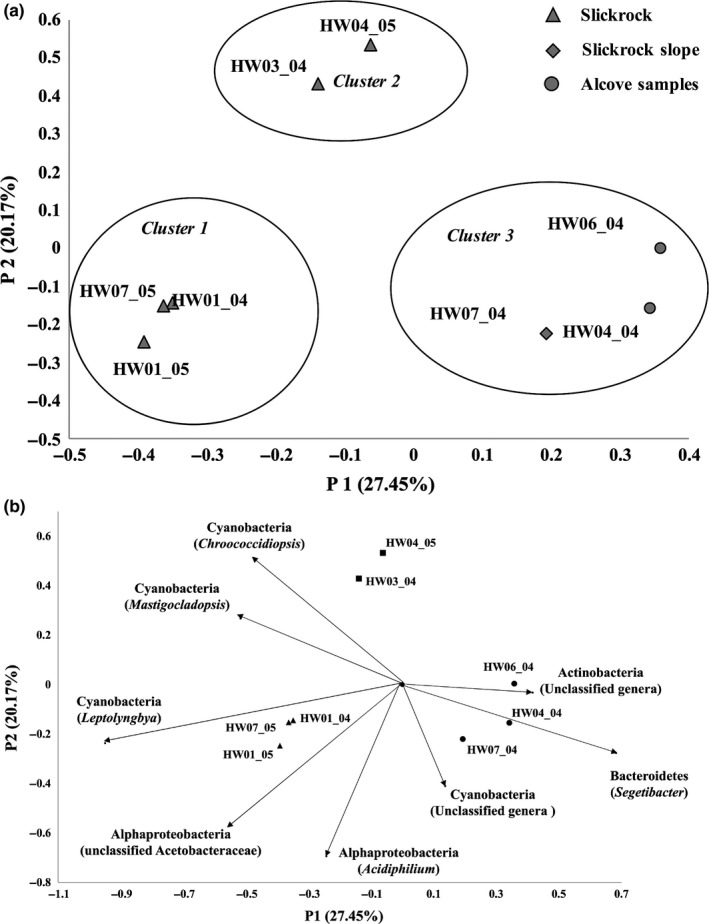
(a) Principal coordinate analysis plot comparing the cryptoendolithic bacterial communities amongst sandstone samples. The *p*‐values obtained using AMOVA test for statistical comparisons of all three clusters were <0.001. (b) Vectors of correlation values from OTUs that explain the clustering of the microbial communities

Richness and diversity indices were calculated for all the sandstone samples based on the number of observed OTUs after all the data were rarefied to normalize the dataset. The resulting observed number of species, inverse Simpson diversity index, and Chao richness indices indicated that the slick rock HW03_04 had the greatest amount of richness and diversity, while HW06_04, the sample from alcove, the least (Table 3). Rarefaction curves based on a 97% similarity showed a considerable difference between the sandstone samples, with HW03_04 showing highest diversity, while HW06_04 exhibited the lowest diversity (data not shown). Based on the principal coordinate analysis (PCoA) plot, the samples separated into three distinct clusters representing cryptoendolithic communities that clustered based on the topography of the sampling sites for the sandstones (Figure 5a). Cluster 1 communities were associated with rock features that were not associated with steep slopes or sediment deposits. Cluster 2 comprised of rock communities that were found near eolian sediment deposits and soils. Cluster 3 communities were associated with steep slopes. The *p*‐values obtained using AMOVA test for statistical comparisons of three clusters, Cluster 1 and 2, Cluster 1 and 3, Cluster 2 and 3, were 0.09, 0.023, and 0.064, respectively. Therefore, the three clusters were significantly different from each other with a *p*‐value <0.001. The vectors of correlation indicated that *Bacteroidetes* and *Actinobacteria* were more prominent in Cluster 3 while *Cyanobacteria* were more dominant in Cluster 1 and Cluster 2 samples (Figure 5b).

**Table 3:** Bacterial diversity metrics based on the 16S rRNA gene analysis of the sandstones samples collected from the GSENM, Utah. Community richness (Chao1 richness estimate), coverage of sampling (in percentage), sobs, and evenness (inverse Simpson diversity index) were calculated in mothur at 97% similarity after normalizing samples to 13041 sequences

Sandstone	Reads	OTUs	Coverage	Sobs	Invsimpson	Chao
HW01_04	20,552	653	98.38	551.80	16.14	832.76
HW01_05	21,250	424	99.03	355.73	14.10	561.33
HW03_04	13,462	750	98.21	742.66	43.06	1007.20
HW04_04	16,243	496	99.08	470.14	33.73	575.92
HW04_05	22,732	657	98.35	528.70	10.18	827.89
HW06_04	13,041	292	99.59	292.00	22.00	326.07
HW07_04	25,046	778	98.40	651.52	22.75	841.60
HW07_05	20,125	489	99.06	439.71	27.43	534.93

### 3.5 Core bacterial communities in cryptoendoliths

**Figure 6:**
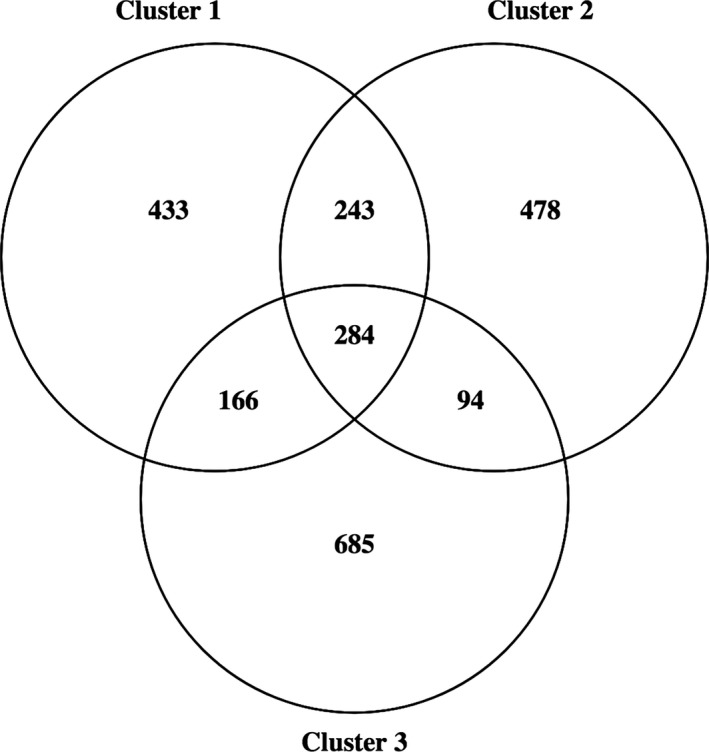
Venn diagram representing the shared OTUs between bacterial communities that indicates the core community existing in the cryptoendoliths in sandstones samples

There was a considerable amount of heterogeneity observed between seemingly similar sites. The total OTUs from all the sandstone samples within each cluster were collated such that each cluster was representative of a group of cryptoendolithic communities associable with varying topography. Comparing the three clusters, it was found that there were 284 OTUs in common, suggesting that a core community exists within the cryptoendolithic habitat (Figure 6). Within this shared group of OTUs, *Cyanobacteria* were the dominant representatives, while *Proteobacteria*, unclassified bacteria, *Actinobacteria,* and *Bacteroidetes* were the next most prevalent phyla (Supporting Information Table S3). *Leptolyngbya* was the major representative of the *Cyanobacteria* along with *Chroococcidiopsis, Mastigocladopsis,* and unclassified Cyanobacteria. Alphaproteobacteria were widely represented by a number of genera including *Acidiphilium*, unclassified Acetobacteraceae*,* unclassified Sphingomonadales, *Sphingomonas*, unclassified Rhizobiales, *Belnapia,* and unclassified Methylobacteriaceae (Supporting Information Table S3). More than half of the shared OTUs were assigned to unclassified genera.

## 4 DISCUSSION

The Jurassic Navajo Sandstone is one of the most porous and permeable sandstone formations found within the GSENM (Chan, Beitler, Parry, Ormo, & Komatsu, 2004) making it a suitable habitat for cryptoendolithic microbes (Kurtz, 2002; Kurtz & Netoff, 2001; Kurtz et al., 2005). Abundance and diversity of cryptoendoliths have been correlated to sandstone color in the past (Bell, 1993; Bell, Athey, & Sommerfeld, 1988). In this study, sandstone color variations did not seem to have not any correlation with the diversity data obtained via next‐generation sequencing, nor did minor differences in the availability of inorganic nitrogen species, phosphate, ferrous iron, or sulfate. pH did not vary considerably between the sandstone samples. Based upon these data, we conclude that these factors have little to no effect on community structure.

Next‐generation sequencing of the sandstone samples revealed the presence of 12 distinct phyla having more than 0.1% abundance in each sample, indicating that this microecosystem is quite diverse. This result was somewhat surprising as a highly diverse community was not expected under these environmental conditions. The number of unclassified sequences strongly suggests that these cryptoendolithic communities harbor a significant number of novel organisms for which there are no data available.

The sequencing data affirm that *Cyanobacteria* is the dominant phylum in cryptoendolithic habitats (Hammes et al., 2013; Kurtz & Netoff, 2001; Kurtz et al., 2005; Lee et al., 2016). Previous studies have reported *Chroococcidiopsis* to be the predominant cyanobacteria observed in arid lithic systems based on morphological characterization (Bell, 1993; Bhatnagar & Bhatnagar, 2005; Büdel & Wessels, 1991; Casamatta et al., 2002; Friedmann, 1980; Pointing & Belnap, 2012; Wessels & Büdel, 1995). However, our data show that *Leptolyngbya* was the most abundant cyanobacterium in this microecosystem followed by unclassified cyanobacteria and *Chroococcidiopsis*. While the overall data indicate that *Leptolyngbya* was the most prevalent genus of cyanobacteria present, there was considerable heterogeneity in cyanobacterial diversity between stone samples.

Bacterial diversity analysis using PCoA plot indicated three distinct clusters of cryptoendolithic communities that correlated to the topography of the sampling sites. From this, we infer that separation of the communities is most likely due to water availability, specifically the duration of time water is present. Cluster 1 communities were only exposed to limited water that may penetrate the pore spaces during a precipitation event with excess precipitation moving downslope as runoff. Cluster 2 communities were potentially exposed to water for longer periods of time as the nearby sediment deposits and soils would tend to hold water in place, making water available via capillary action. Cluster 3 communities were exposed to water as it percolated downslope from higher elevations through pores and cracks in the sandstone. The vectors of correlation indicated that the microbial communities with longer water availability tended to have more *Bacteroidetes* and *Actinobacteria,* while *Cyanobacteria* were dominant under less favorable conditions with limited water availability. From this analysis, we conclude that water availability is one of the primary forces affecting community structure. This conclusion is in concurrence with a current thought regarding the effects of episodic events such as precipitation on microbial communities (Meslier et al., 2018; Nielsen & Ball, 2015).

The sandstone samples from alcoves, HW04_04 and HW06_04, had the least microbial diversity as compared to other samples, including sample HW07_04, which clustered with these samples. This suggests that the presence of water for an extended period of time is enough to allow a small group to outcompete other microbes within the slick‐rock core. The two alcove samples had considerably higher *Bacteroidetes* and *Deinococcus‐Thermus* outcompeting the cyanobacterial members. Given the diversity within the *Bacteroidetes* phylum and the lack of information associated with the unclassified OTUs, it is not possible to draw a specific conclusion regarding the underlying driving factors resulting in these shifts in community structure. Previous studies suggest that a reduction in temperature and water stress brings a marked shift in the endolithic community (Bell et al., 1988). HW07_04, slick rock slope sample, and HW06_06, alcove sample, harbored *Actinomycetes* in slightly more abundance than other sandstones and also exhibited the highest percentage of bacteria assigned to unclassified bacteria amongst all the samples.

When we examine the diversity shared between the clusters obtained via PCoA plot, we find an overlapping core of 284 OTUs that represents the core microbial community ubiquitous in sandstone samples collected during different years and topographic locations. *Cyanobacteria* were the most abundant in terms of the cyanobacterial OTUs being observed the maximum number of times amongst all the shared diversity. Nearly half of the shared OTUs belong to unclassified genera, indicating the extent of unexplored diversity in this microbial ecosystem. Thirty percent of the shared diversity belonged to Alphaproteobacteria with Acetobacteraceae being the dominant members in this phylum. In the context of the overall community, these bacteria are subsisting on the exudates of the dominant cyanobacteria. Given that the genus *Acidiphilium* comprises a large proportion of the Acetobacteraceae, with members of this genus known to be capable of aerobic iron reduction, we hypothesize that these bacteria are integral members of these cryptoendolithic communities whose role is to reduce ferric iron to ferrous iron (Bilgin, Silverstein, & Jenkins, 2004; Bridge & Johnson, 2000). The dominant cyanobacteria are slow‐growing and desiccation‐resistant due to their ability to produce organic‐rich extracellular polymeric substances (Ferris & Lowson, 1997). These extracellular polymers can be subsequently metabolized by heterotrophic bacteria, lowering the ambient pH values through the production of low‐molecular‐weight organic acids and respiratory carbon dioxide (Ferris & Lowson, 1997; Gorbushina, 2007). This basic set of metabolic processes set the conditions required for the aerobic reduction of iron by *Acidiphilium* spp., which will return the pH back to neutrality (Bilgin et al., 2004; Kusel, Dorsch, Acker, & Stackebrandt, 1999). Under these conditions, the reduced iron would be captured by the EPS produced by members of these communities (Hammes et al., 2013). These observations allow us to outline a hypothetical ecological cycle where the cyanobacteria produce EPS and other metabolites that support a robust heterotrophic community. General metabolic processes cause a localized lowering of pH, which, in combination with the metabolites produced by *Cyanobacteria*, supports the growth of *Acidiphilium* spp. that reduces ferric iron to ferrous iron, making it more readily available to the larger community.

Comparing these communities to other desert communities, we see that with only a few exceptions, namely the Negev Desert and the Atacama Desert (Bell, 1993; Connon, Lester, Shafaat, Obenhuber, & Ponce, 2007; Crits‐Christoph et al., 2013; Friedmann, Lipkin, & Ocampo‐Paus, 1967; Wierzchos et al., 2012), the microbial diversity is quite similar (Antony et al., 2012; Bell, Athey, & Sommerfeld, 1986; de la Torre et al., 2003; Friedmann, 1980; Lacap‐Bugler et al., 2017). In comparison with the community structure of local desert soils, we find that the overall structure is comparable, with *Cyanobacteria* and *Proteobacteria* numbers being higher on a relative basis (Garcia‐Pichel, Johnson, Youngkin, & Belnap, 2003; Garcia‐Pichel, Lopez‐Cortes, & Nubel, 2001; Lee et al., 2016). However, we also note that while the structure is similar, the presence of certain phyla such as the *Acidobacteria, Verrucomicrobia,* and *Planctomycetes* is more pronounced in the soil environments (Gundlapally & Garcia‐Pichel, 2006; Nagy, Pérez, & Garcia‐Pichel, 2005). This similarity in community structure is not unexpected as the proximity of the soil and cryptoendolithic communities logically suggests that one of these two distinct ecosystems influences the assembly of the other. Wind dispersal of dust and sand in semiarid regions has the potential to move not only sediments, but also bacterial cells from one habitat to the other. Thus, we cannot say with certainty which community influences the assembly of the other. However, the differences in diversity between the cryptoendolithic community and the soil community can be attributed to more moderate conditions within the soil, specifically higher nutrient levels, longer availability of water, and the presence of reduced carbon. While wind dispersal of cells cannot be attributed to the colonization of newly deposited sediments or fresh stone surfaces in a directional manner, water dispersal of cells is most likely from the cryptoendolithic community to the soil and sediment communities. The sandstone outcrops sampled in this study have very little sediment cover and are generally higher in elevation than the surrounding soils. Thus, when precipitation runs downslope, cells and sediment will be carried from the cryptoendolithic communities to the local soils and sediment deposits.

This research provides evidence that unexplored cryptoendolithic bacterial diversity exists in the Jurassic Navajo sandstones, thereby identifying a conservation value for these desert communities. The cyanobacterial diversity in cryptoendolithic communities varies with location, potentially reflecting their adaptation to available moisture regimes in this semiarid ecosystem. Further studies are required to expand the understanding of the biological functions of the cryptoendolithic communities in sandstones.

## CONFLICT OF INTEREST

None declared.
